# Deep Learning-Assisted MRI for Differentiating Parotid Gland Tumors: Comparison with FNAB and Radiologic Assessment

**DOI:** 10.3390/diagnostics16162577

**Published:** 2026-08-15

**Authors:** Servet Erdemes, Ömer Türk, Mahmut Ağırtmış, Recep Aydın

**Affiliations:** 1 Department of Otorhinolaryngology, Harran University Faculty of Medicine, Şanlıurfa 63290, Türkiye; 2 Department of Computer Engineering, Faculty of Engineering and Architecture, Mardin Artuklu University, Mardin 47100, Türkiye; 3 Department of Radiology, Mehmet Akif İnan Training and Research Hospital, Şanlıurfa 63300, Türkiye

**Keywords:** artificial intelligence, deep learning, magnetic resonance imaging, parotid gland tumors, fine-needle aspiration biopsy

## Abstract

**Background/Objectives:** To evaluate the diagnostic performance of an MRI-based deep learning (DL) model for differentiating benign and malignant parotid gland tumors and to compare its performance with radiologic assessment and fine-needle aspiration biopsy (FNAB), using histopathology as the reference standard. **Methods:** This retrospective single-center study included 144 consecutive patients with histopathologically confirmed parotid gland tumors who underwent parotidectomy between January 2020 and December 2024. Preoperative MRI examinations were analyzed using a ResNet50-based convolutional neural network incorporating a Convolutional Block Attention Module (CBAM). Model performance was evaluated using patient-level stratified 5-fold cross-validation and compared with MRI and FNAB using sensitivity, specificity, accuracy, positive predictive value (PPV), and negative predictive value (NPV). **Results:** The DL model achieved a sensitivity of 79.3% (95% CI, 61.6–90.2), specificity of 93.9% (95% CI, 87.9–96.9), and an overall accuracy of 91.0% (95% CI, 85.3–94.5). MRI demonstrated a sensitivity of 86.2% and specificity of 93.0%, whereas FNAB achieved a sensitivity of 82.8% and specificity of 96.5%. The DL model showed consistent performance across the validation folds. **Conclusions:** MRI-based deep learning demonstrated high diagnostic performance for the preoperative classification of parotid gland tumors and may serve as a complementary decision-support tool alongside MRI and FNAB. Although promising, these findings are limited by the retrospective single-center design and the lack of external validation. Prospective multicenter studies are required before routine clinical implementation.

## 1. Introduction

Parotid gland tumors represent approximately 70–80% of all salivary gland neoplasms and comprise a heterogeneous spectrum of benign and malignant lesions with diverse histopathological characteristics [1]. Accurate preoperative discrimination between these lesions remains clinically challenging because their presenting symptoms, growth patterns, and imaging characteristics frequently overlap [2]. Establishing the correct diagnosis before surgery is essential for selecting the appropriate surgical strategy, determining the extent of resection, preserving facial nerve function, and optimizing postoperative management [3].

Fine-needle aspiration biopsy (FNAB) is widely accepted as the initial diagnostic procedure for parotid gland lesions owing to its minimally invasive nature, relatively low cost, and broad availability. Nevertheless, the diagnostic performance of FNAB is influenced by factors such as sampling adequacy, tumor heterogeneity, and the experience of the cytopathologist. Previous studies have reported sensitivity rates ranging from 80% to 90%, whereas specificity generally exceeds 90% [4,5]. Although FNAB provides valuable cytological information, indeterminate or false-negative results may occasionally complicate clinical decision-making.

Magnetic resonance imaging (MRI) has become the preferred imaging modality for evaluating parotid gland tumors because of its superior soft-tissue contrast and excellent multiplanar capability [6]. Conventional MRI sequences, including T1-weighted, T2-weighted, and diffusion-weighted imaging (DWI), provide important morphological and functional information that contributes to lesion characterization. However, considerable overlap in imaging findings between benign and malignant tumors often limits diagnostic confidence when MRI is interpreted alone [3]. Beyond lesion characterization, MRI also provides valuable information regarding tumor extent, deep-lobe involvement, perineural spread, parapharyngeal extension, and the relationship of the lesion to adjacent neurovascular structures, thereby facilitating preoperative planning and surgical decision-making [7].

In recent years, artificial intelligence (AI), particularly deep learning (DL), has emerged as a promising approach for medical image analysis. Deep convolutional neural networks (CNNs) can automatically identify complex imaging patterns that may not be readily appreciated by visual inspection, offering the potential to improve diagnostic accuracy, reproducibility, and observer independence [8]. Several MRI-based deep learning models have demonstrated encouraging results in differentiating benign and malignant parotid gland tumors [9,10,11]. Likewise, the integration of deep learning with radiomic analysis of ultrasound images has yielded excellent diagnostic performance, including successful validation in external datasets [12]. Collectively, these findings indicate that AI-assisted image analysis has the potential to complement conventional diagnostic methods by providing objective and standardized image interpretation.

Despite these advances, current evidence remains limited because most published studies have evaluated deep learning models independently or compared them with only a single conventional diagnostic modality. Direct comparisons of MRI-based deep learning, routine radiologic assessment, and FNAB within the same patient cohort remain scarce. Consequently, the incremental clinical value of deep learning in routine preoperative assessment has not yet been clearly established.

Therefore, the primary objective of the present study was to compare the diagnostic performance of MRI-based deep learning, conventional radiologic assessment, and FNAB for differentiating benign and malignant parotid gland tumors using histopathological diagnosis as the reference standard. The principal contribution of this study lies not in proposing a novel neural network architecture, but in evaluating an attention-enhanced convolutional neural network alongside two established preoperative diagnostic approaches within the same patient cohort. We also aimed to investigate whether MRI-based deep learning could serve as a reproducible, non-invasive, and observer-independent decision-support tool that complements conventional radiologic assessment and FNAB.

## 2. Materials and Methods

### 2.1. Study Design and Patient Selection

This retrospective single-center observational study included consecutive patients who underwent parotidectomy at the Department of Otorhinolaryngology of Harran University Faculty of Medicine between January 2020 and December 2024. Only patients with a definitive postoperative histopathological diagnosis of a parotid gland tumor were considered eligible for inclusion.

Patients were included if they met all of the following criteria: (1) availability of preoperative magnetic resonance imaging (MRI) performed according to the institutional imaging protocol; (2) availability of preoperative fine-needle aspiration biopsy (FNAB) results; and (3) complete postoperative histopathological confirmation. Patients were excluded if MRI examinations were unavailable or of insufficient quality for image analysis, if FNAB results were missing, if histopathological confirmation was unavailable, or if clinical data were incomplete.

A total of 144 consecutive patients fulfilled the eligibility criteria and were included in the final analysis. Because of the retrospective design, no formal sample size calculation was performed before patient selection. Instead, the study cohort consisted of all eligible consecutive patients treated during the predefined study period, thereby minimizing selection bias. The final sample size was comparable to those reported in previous single-center deep learning studies on parotid gland tumors. In addition to the tumor cohort, a separate control cohort consisting of 122 individuals without parotid gland pathology was included for the development and evaluation of the three-class deep learning model. These individuals had undergone neck MRI for clinical indications unrelated to the parotid glands, and their MRI examinations showed no evidence of parotid gland lesions. All control examinations were acquired at the same institution using the same 1.5-T Siemens MRI scanner and standardized imaging protocol as the tumor examinations (Siemens Healthineers, Forchheim, Germany). The control subjects were not part of the 144-patient tumor cohort and were not included in the direct clinical comparison of deep learning, radiologic assessment, and FNAB.

### 2.2. Ethical Approval

This study was approved by the Clinical Research Ethics Committee of Harran University Faculty of Medicine (Approval No: HRÜ/24.16.09, Date: 21 October 2024), and all procedures were performed in accordance with the ethical standards of the institutional and/or national research committee and the 1964 Declaration of Helsinki and its later amendments.

### 2.3. Imaging Protocol

All MRI examinations were performed using the same 1.5-T Siemens MRI scanner with a standardized institutional imaging protocol. The institutional MRI protocol remained unchanged throughout the entire study period from January 2020 to December 2024. The protocol included axial and coronal T1-weighted, T2-weighted, diffusion-weighted imaging (DWI), fat-suppressed T1-weighted pre-contrast, and fat-suppressed T1-weighted post-contrast sequences for all patients. Images obtained from all included MRI sequences and imaging planes were used in the deep learning analysis. The coronal MRI image presented in the manuscript was selected solely as a representative visual example and does not indicate that the analysis was restricted to coronal images or to a single MRI sequence. MRI provides excellent soft-tissue contrast and comprehensive anatomical information, enabling assessment of tumor extent, deep-lobe involvement, perineural spread, parapharyngeal extension, and the relationship of the lesion to adjacent neurovascular structures, thereby supporting preoperative surgical planning [7]. Conventional MRI interpretation was performed independently by a board-certified head and neck radiologist with more than 10 years of experience, who was blinded to both FNAB findings and the final histopathological diagnosis throughout the image evaluation process.

### 2.4. Fine-Needle Aspiration Biopsy (FNAB)

Fine-needle aspiration biopsy (FNAB) procedures were performed under ultrasound guidance using a 23-gauge needle by an experienced radiologist. Cytological samples were evaluated in the cytopathology laboratory and reported according to the Milan System for Reporting Salivary Gland Cytopathology [13]. The Milan System comprises seven diagnostic categories: (I) nondiagnostic, (II) nonneoplastic, (III) atypia of undetermined significance (AUS), (IVa) benign neoplasm, (IVb) salivary gland neoplasm of uncertain malignant potential (SUMP), (V) suspicious for malignancy, and (VI) malignant. This system provides a standardized framework for estimating malignancy risk and guiding clinical management. For diagnostic performance analysis, categories II and IVa were considered benign, whereas categories V and VI were classified as malignant. Categories I (nondiagnostic), III (AUS), and IVb (SUMP) were prespecified as indeterminate categories that would not be included in binary diagnostic performance calculations. However, no patients in the present cohort were assigned to these categories; therefore, all 144 tumor patients were included in the FNAB performance analysis. Importantly, FNAB results that were discordant with the final histopathological diagnosis were not excluded from the analysis, in order to reflect real-world diagnostic performance. Histopathological findings obtained after parotidectomy were used as the reference standard for all diagnostic comparisons.

### 2.5. Deep Learning Model and Analysis

Deep learning analysis was performed using a convolutional neural network (CNN) based on the ResNet50 architecture integrated with a Convolutional Block Attention Module (CBAM). The CBAM enhances feature representation by sequentially applying channel and spatial attention mechanisms, enabling the network to focus on diagnostically relevant image regions while suppressing less informative features.

The model was designed as a three-class classifier, generating probability outputs for benign tumors, malignant tumors, and normal controls. Inclusion of a normal control class enabled the network to distinguish physiological parotid anatomy from tumor-containing images and was intended to reduce false-positive classifications.

MRI examinations were retrieved in DICOM format and subjected to a standardized preprocessing pipeline before model development. Images were anonymized, resized to 224 × 224 pixels, and normalized using the ImageNet mean and standard deviation values. On-the-fly data augmentation was applied exclusively to the training folds to improve model robustness and reduce overfitting. The transformations were deliberately restricted to small random rotations (±10°), horizontal flipping, translations (±5%), and mild intensity variations. Small rotations and translations were intended to simulate minor differences in patient positioning and image framing while preserving the underlying lesion morphology. Horizontal flipping was considered appropriate because lesion laterality was not a classification target and both the right and left parotid glands were represented in the dataset. Mild intensity variation was used to improve robustness to modest acquisition-related signal differences. Training accuracy was calculated at the image level, with each selected MRI image treated as an individual sample. In contrast, validation accuracy was calculated at the subject level by aggregating all image-level predictions belonging to the same subject using majority voting. This aggregation reduced the influence of individual misclassified images and provided a more stable subject-level validation estimate.

Representative MRI images were manually selected from all included MRI sequences and imaging planes by a single experienced head and neck radiologist. No fixed numerical quota was applied to individual MRI sequences or imaging planes; image selection was based on the predefined anatomical and morphological criteria rather than on equal sequence representation. This strategy was chosen to prioritize diagnostically informative tumor representation rather than artificial numerical balance across sequences. Image selection was based on predefined anatomical and morphological criteria, including visualization of the lesion at different anatomical levels, the maximum tumor diameter, tumor margins, internal heterogeneity, and adjacent anatomical structures. To standardize the manual image-selection process, the same predefined anatomical and morphological criteria were applied consistently to all subjects. The number of selected images was not identical for every subject because the extent, size, and morphological characteristics of the lesions varied among patients. Because the number of patients with malignant tumors was substantially lower than the number of patients with benign tumors, a greater number of original representative MRI images was selected from malignant cases to reduce image-level class imbalance and to provide broader representation of malignant tumor morphology. The final image dataset consisted of 528 malignant, 780 benign, and 734 normal control MRI images obtained from 29 patients with malignant tumors, 115 patients with benign tumors, and 122 control individuals, respectively. These image counts represent original MRI images obtained across multiple sequences and anatomical levels and do not represent independent subjects or augmented samples.

All images belonging to the same individual were assigned exclusively to a single cross-validation fold. Therefore, no individual contributed images to both the training and validation datasets. Augmented images were not counted as original images or included as independent validation samples. The model generated an individual three-class prediction for each selected MRI image. To obtain a single subject-level classification, all image-level predictions belonging to the same subject were aggregated using majority voting. The class receiving the highest number of image-level votes was assigned as the final subject-level prediction. This aggregation was performed separately for the subjects in each validation fold, using only the predictions generated while the corresponding subject was included in the validation fold. The direct clinical comparison of deep learning, radiologic assessment, and FNAB was performed at the patient level using the 144 patients with histopathologically confirmed parotid gland tumors. For this binary clinical comparison, malignant predictions were considered positive, whereas benign and control predictions were grouped as non-malignant. The separate cohort of 122 normal controls was included only in the three-class deep learning evaluation and was not included in the comparison with MRI and FNAB.

Model performance was assessed using patient-level stratified five-fold cross-validation. For each iteration, four folds were used for model training and one fold for validation. Class imbalance was addressed using weighted cross-entropy loss, with class weights determined according to the inverse frequency of each class. Optimization was performed using the Adam optimizer with an initial learning rate of 1 × 10^−4^, and model convergence was monitored using early stopping and learning-rate scheduling.

**Figure 1:**
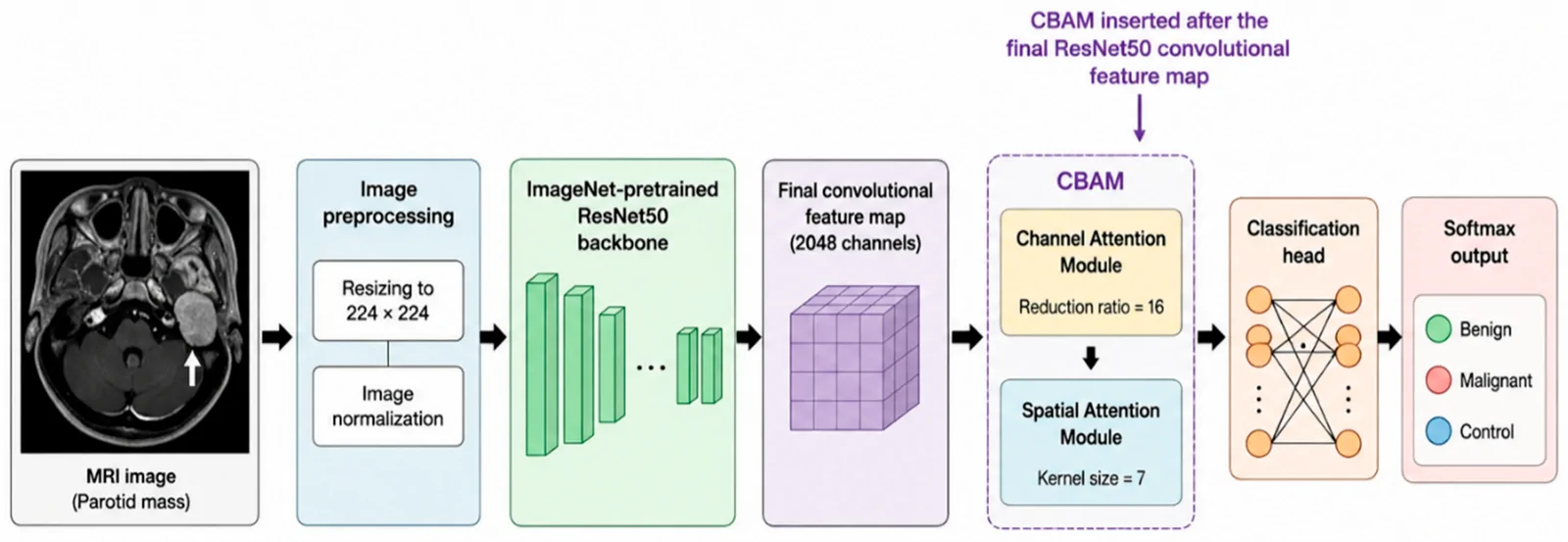
Architecture of the proposed ResNet50+CBAM framework. The CBAM was inserted after the final 2048-channel convolutional feature map of the ResNet50 backbone. Channel attention was applied first, using a reduction ratio of 16, followed by spatial attention with a kernel size of 7. The refined feature representation was subsequently passed to the classification head to generate benign, malignant, and control class probabilities.

A CBAM was inserted immediately after the final 2048-channel convolutional feature map of the ResNet50 backbone and before the final classification stage. Within CBAM, channel attention was applied first using a reduction ratio of 16, followed by spatial attention with a kernel size of 7, consistent with the original CBAM implementation [14]. The detailed position and internal sequence of the CBAM are illustrated in Figure 1.

Images were processed in mini-batches of 16, and each fold was trained for 10 epochs using the CrossEntropyLoss function. No additional hyperparameter optimization was performed in order to preserve architectural reproducibility and facilitate comparison with previously published studies.

ResNet50 was selected as the backbone architecture because its residual connections facilitate stable optimization of deep networks, while its ImageNet-pretrained weights provide a practical transfer-learning framework for a moderately sized medical imaging dataset [15]. In addition, its modular structure allows straightforward integration of the CBAM attention mechanism without substantially altering the underlying feature-extraction pathway. The purpose of incorporating CBAM was to refine the final convolutional feature maps through sequential channel and spatial attention rather than to introduce an entirely new neural network architecture.

To assess the suitability of the selected architecture, comparative experiments were performed using the baseline ResNet50, DenseNet121, and EfficientNet-B0 models. All architectures were evaluated using identical image preprocessing procedures, subject-level five-fold cross-validation partitions, optimization settings, and training conditions.

Model performance during training was evaluated using slice-level accuracy, precision, recall, and F1-score. However, because MRI interpretation and FNAB are performed at the patient level in clinical practice, all diagnostic comparisons with MRI and FNAB were conducted using patient-level predictions. Diagnostic performance was quantified using sensitivity, specificity, accuracy, positive predictive value (PPV), and negative predictive value (NPV), with histopathological diagnosis serving as the reference standard.

### 2.6. Statistical Analysis

All statistical analyses were performed using IBM SPSS Statistics version 26 and MedCalc version 20. Descriptive data were summarized as mean ± standard deviation, median and interquartile range (IQR), or number and percentage, as appropriate. Diagnostic performance metrics, including sensitivity, specificity, accuracy, positive predictive value (PPV), and negative predictive value (NPV), were calculated for radiologic MRI assessment, FNAB, and the deep learning model. For each metric, 95% confidence intervals (CIs) were calculated using the Wilson score method. Receiver operating characteristic (ROC) curve analysis was performed for all three diagnostic approaches, and the area under the curve (AUC) was calculated. Pairwise comparisons of the ROC curves—MRI versus FNAB, MRI versus deep learning, and FNAB versus deep learning—were performed using the DeLong test. A *p* value of < 0.05 was considered statistically significant.

## 3. Results

The study included 144 patients with histopathologically confirmed parotid gland lesions. The mean ± SD age was 44.9 ± 18.0 years, with a median of 48.0 years (interquartile range, 30.0–59.5; range, 6–77 years). There were 80 males (55.6%) and 64 females (44.4%). Lesions were located on the left side in 82 patients (56.9%) and on the right side in 62 patients (43.1%). According to histopathological diagnosis, 115 (79.9%) lesions were benign and 29 (20.1%) were malignant. Among benign tumors, pleomorphic adenoma was the most common histologic subtype (71 patients, 49.3%), followed by Warthin tumor (44 patients, 30.6%). Among malignant tumors, mucoepidermoid carcinoma (23 patients, 16.0%) and adenoid cystic carcinoma (6 patients, 4.2%) were the leading histologic subtypes (Table 1).

The tumor cohort consisted of 144 patients, including 115 patients with benign tumors and 29 patients with malignant tumors. A separate cohort of 122 individuals without parotid gland pathology was included as the normal control group for the three-class deep learning analysis. The final image dataset comprised 780 benign, 528 malignant, and 734 control MRI images. These image counts represent original MRI images obtained across multiple sequences and anatomical levels rather than independent subjects. The subject- and image-level composition of the dataset is summarized in Table 1.

**Figure 2:**
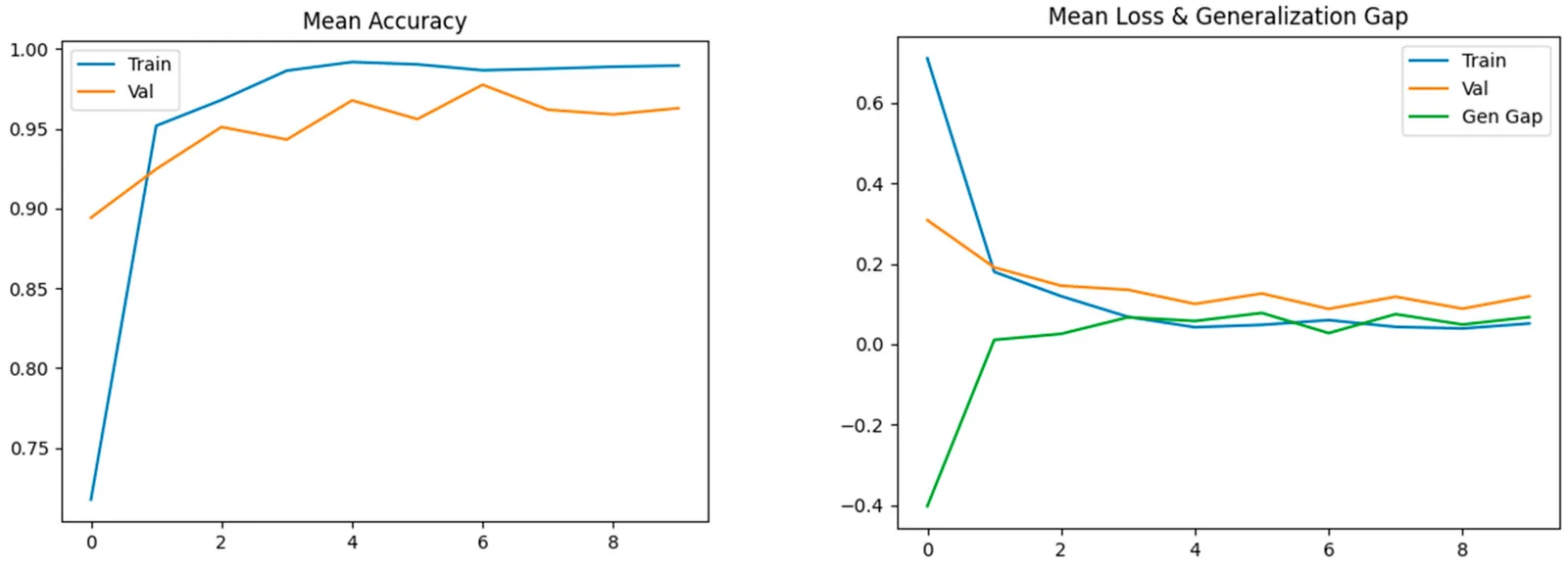
Mean training and validation accuracy and loss curves across the five subject-level cross-validation folds. Training accuracy was calculated at the image level, whereas validation accuracy was calculated at the subject level by majority voting across all selected MRI images belonging to each subject.

Across the five subject-level cross-validation folds, the ResNet50+CBAM model achieved a mean image-level classification accuracy of 0.9716 ± 0.0127. The mean training and validation accuracy and loss curves obtained across all folds are presented in Figure 2.

During the initial epochs, the mean validation accuracy was higher than the mean training accuracy. This pattern was primarily attributable to the use of ImageNet-pretrained weights and the different levels at which training and validation accuracy were calculated. Training accuracy was calculated at the image level, whereas validation accuracy was determined at the subject level by majority voting across all selected images belonging to each subject. Majority voting reduces the influence of incorrectly classified individual images and can therefore produce a more stable and higher validation accuracy, particularly during the initial epochs. Thus, the observed difference was not attributed primarily to data augmentation or class weighting. As training progressed, the training and validation curves converged and remained relatively stable without marked progressive divergence.

Comparative benchmarking was performed to evaluate the selection of the ResNet50+CBAM architecture. The baseline ResNet50, DenseNet121, and EfficientNet-B0 models were evaluated using the same preprocessing pipeline, subject-level five-fold cross-validation partitions, optimization settings, and training conditions. The comparative image-level performance results are presented in Table 2.

Although model training performance was evaluated using slice-level data, the clinical diagnostic performance of the model was assessed at the patient level to ensure comparability with MRI and FNAB results.

Among the evaluated CNN architectures, ResNet50+CBAM achieved the highest mean accuracy, precision, recall, and F1-score. Class-wise analysis also demonstrated consistently high performance across the benign, malignant, and control classes. In the component-level ablation comparison, adding CBAM to the baseline ResNet50 increased the mean image-level accuracy from 0.86 to 0.9716, precision from 0.74 to 0.9747, recall from 0.80 to 0.9723, and F1-score from 0.76 to 0.9731 under identical preprocessing, cross-validation partitions, and training conditions. These numerical improvements support the contribution of attention-guided feature refinement to the observed classification performance.

**Figure 3:**
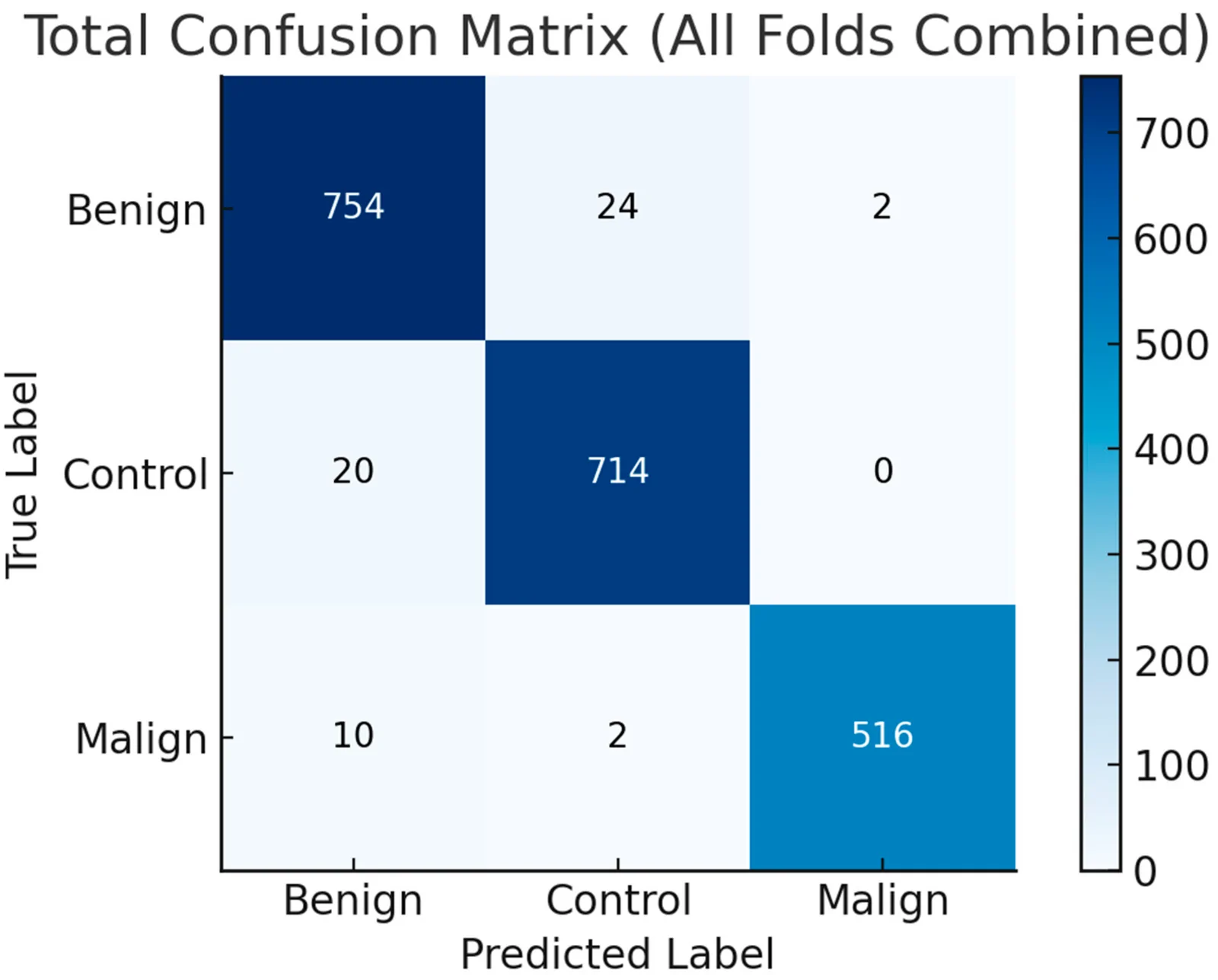
Combined image-level confusion matrix across the five subject-level cross-validation folds.

The combined image-level confusion matrix obtained by aggregating the predictions across the five validation folds is presented in Figure 3.

As shown in Figure 3, among the 528 malignant images, 516 were correctly classified, while 10 were misclassified as benign and 2 as control. Of the 780 benign images, 754 were correctly classified, 24 were misclassified as control, and 2 as malignant. Finally, among the 734 control images, 714 were correctly classified, whereas 20 were misclassified as benign.

**Figure 4:**
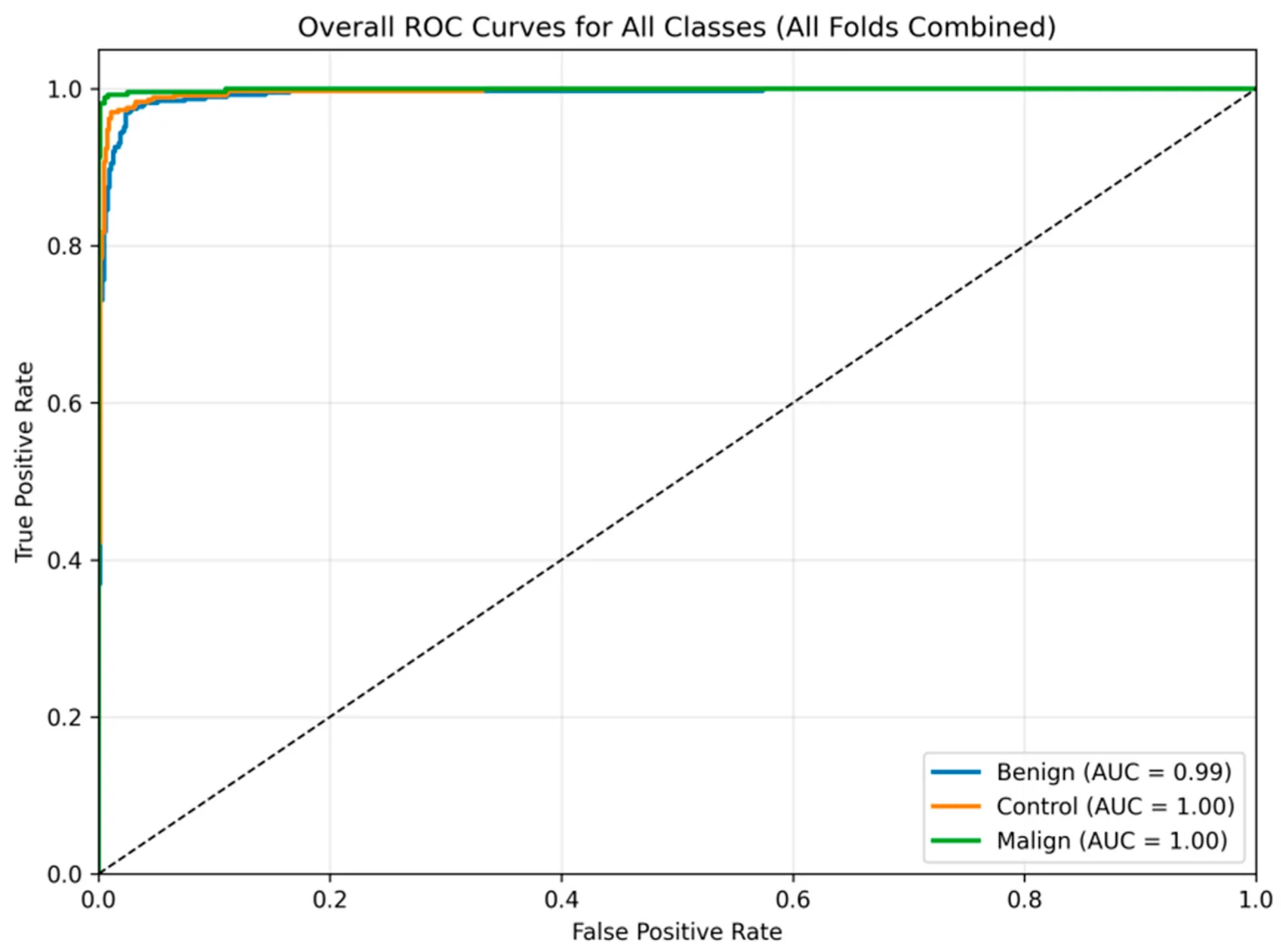
Image-level one-vs.-rest ROC curves of the three-class ResNet50+CBAM model. The ROC curves were generated using out-of-fold image predictions pooled across the five subject-level cross-validation folds. The AUC values were 0.99 for the benign class, 1.00 for the malignant class, and 1.00 for the control class.

Image-level one-vs.-rest ROC curves for the benign, malignant, and control classes of the three-class deep learning model are presented in Figure 4.

At the image level, the one-vs.-rest AUC values were 0.99 for benign tumors and 1.00 for both malignant tumors and normal controls. These values reflect the performance of the three-class deep learning model at the image level and should not be directly compared with the patient-level binary AUC values obtained in the clinical comparison of deep learning, MRI, and FNAB.

**Figure 5:**
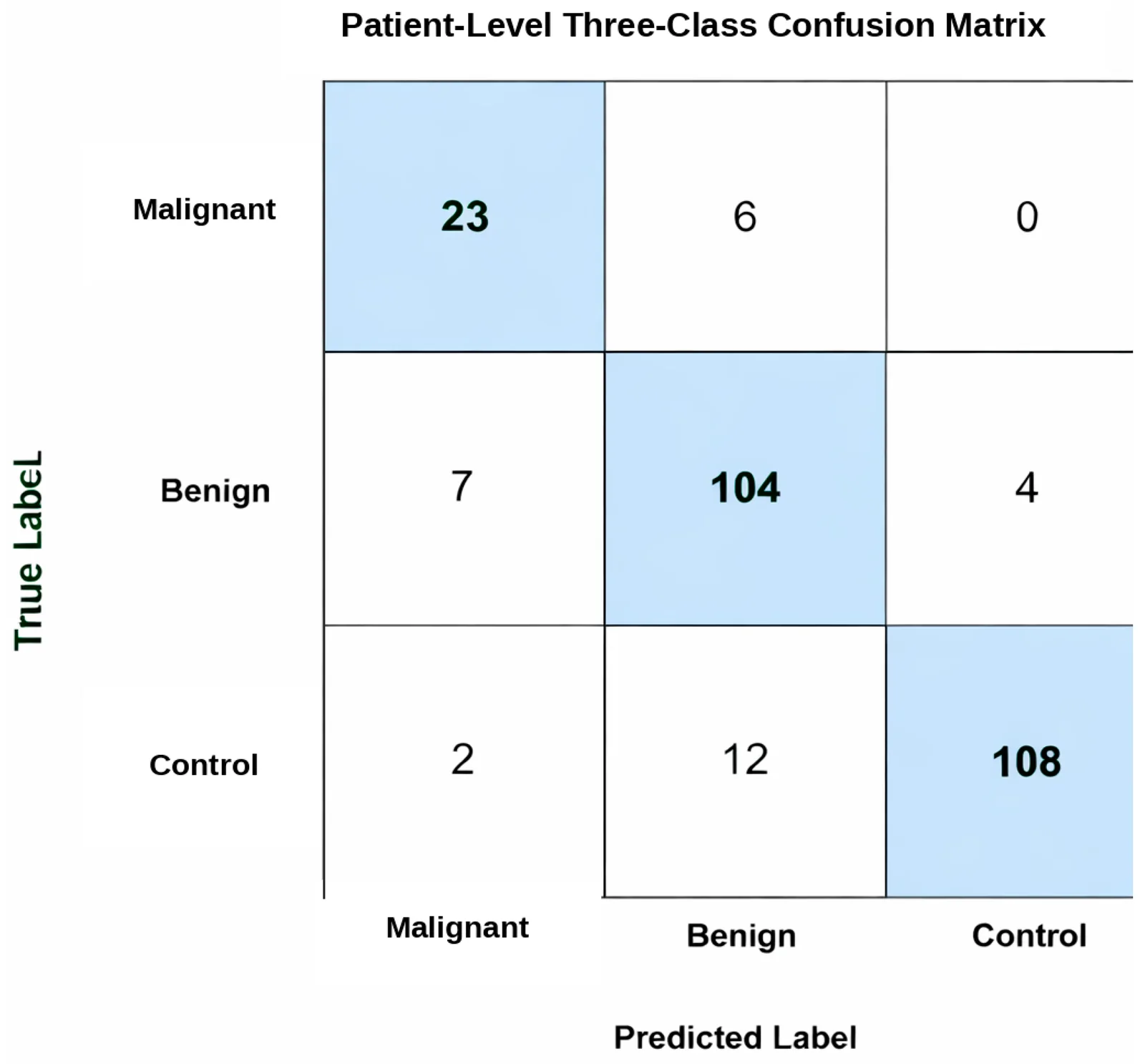
Patient-level three-class confusion matrix of the ResNet50+CBAM model. The matrix shows subject-level predictions for malignant tumors, benign tumors, and normal controls.

Subject-level predictions were obtained by majority voting across all selected MRI images belonging to each subject. The resulting three-class confusion matrix is presented in Figure 5.

At the patient level, the model correctly classified 23 of 29 malignant tumors, 104 of 115 benign tumors, and 108 of 122 normal controls. Among malignant tumors, six were classified as benign and none as control. Among benign tumors, seven were classified as malignant and four as control. Of the normal controls, two were classified as malignant and 12 as benign.

### Grad-CAM Analysis

**Figure 6:**
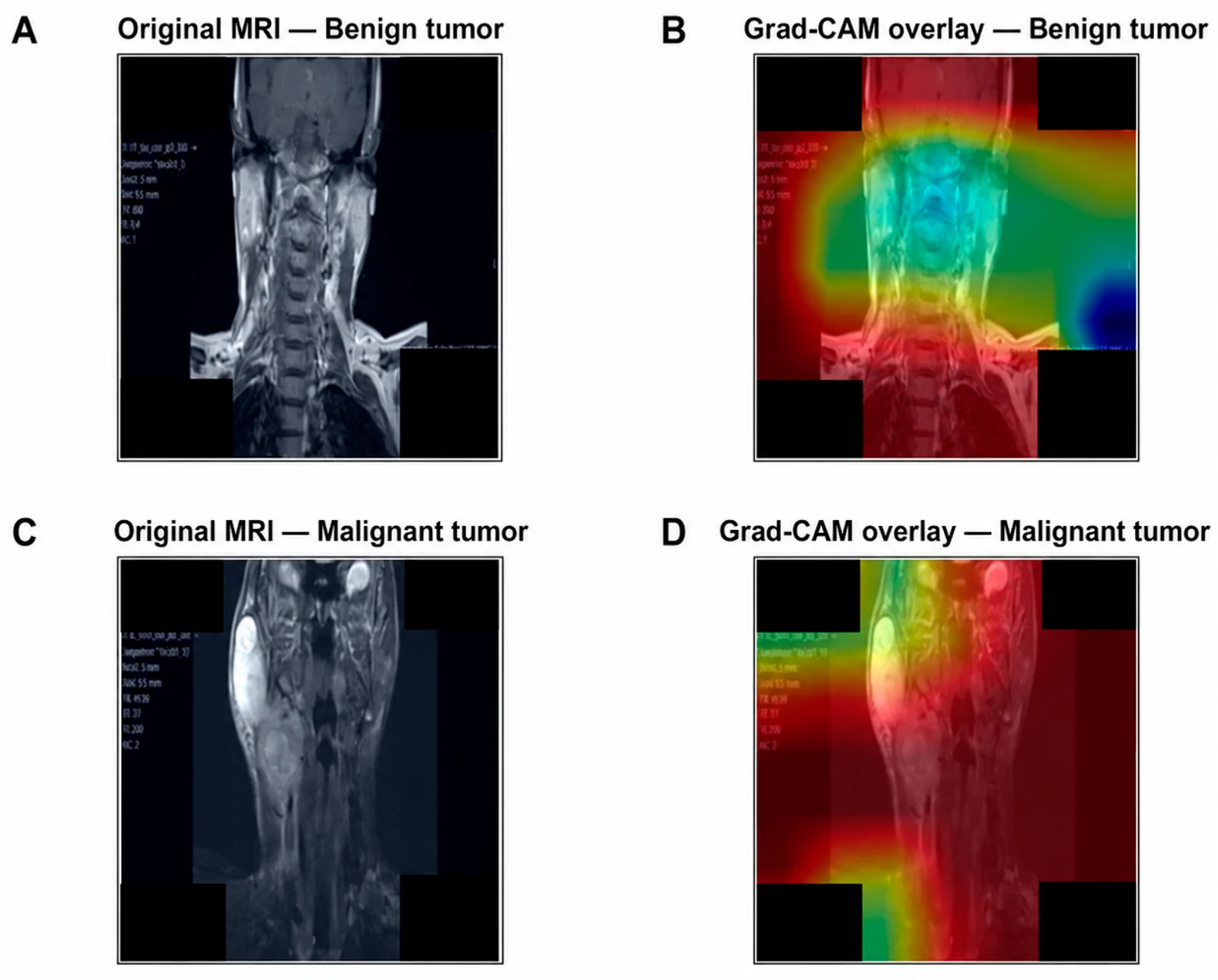
Representative Grad-CAM visualizations of benign and malignant parotid tumors. Panels (**A**,**C**) show the original MRI images of representative benign and malignant tumors, respectively, with the lesions indicated by arrows. Panels (**B**,**D**) show the corresponding Grad-CAM overlays. Warmer colors indicate image regions with greater contribution to the model prediction. Grad-CAM was used for qualitative interpretability and should not be considered a tumor segmentation or localization method.

Grad-CAM was used to provide a qualitative visualization of the image regions contributing to the model predictions, with representative examples shown in Figure 6. In the representative benign and malignant cases, the activation maps showed partial spatial correspondence with the parotid tumor regions. However, activation was not restricted exclusively to the lesions, and some attention was also observed in adjacent anatomical structures. Therefore, these visualizations should be interpreted as qualitative explanations of model attention rather than precise tumor localization or segmentation results.

Table 3 summarizes the patient-level diagnostic performance of MRI, FNAB, and the deep learning model using histopathology as the reference standard; additional details are provided in  Supplementary Table S1. The deep learning model achieved a sensitivity of 79.3% (95% CI: 61.6–90.2) and a specificity of 93.9% (95% CI: 87.9–96.9), with an overall accuracy of 91.0% (95% CI: 85.3–94.5). The positive predictive value was 76.7% (95% CI: 58.8–88.2), while the negative predictive value reached 94.7% (95% CI: 89.0–97.5). In the patient-level binary analysis of the 144 tumor patients, the AUC values were 0.91 for conventional MRI assessment, 0.94 for FNAB, and 0.99 for the deep learning model. Pairwise DeLong comparisons showed differences between deep learning and MRI (*p* < 0.001), deep learning and FNAB (*p* = 0.002), and FNAB and MRI (*p* = 0.041). These patient-level binary AUC values are distinct from the image-level one-vs.-rest AUC values reported for the three-class deep learning model in Figure 4 and Table 2.

## 4. Discussion

The principal contribution of the present study is the direct comparison of MRI-based deep learning, conventional radiologic assessment, and fine-needle aspiration biopsy (FNAB) within the same patient cohort using histopathological diagnosis as the reference standard. The study was not designed to introduce a novel neural network architecture; rather, it evaluated whether the established ResNet50 architecture, enhanced with a CBAM attention module, could provide clinically relevant diagnostic support when compared with conventional preoperative diagnostic methods. The present study demonstrated that the proposed deep learning model achieved diagnostic performance comparable to established preoperative diagnostic methods and may offer a non-invasive, standardized, and observer-independent image analysis approach. Rather than replacing MRI interpretation or FNAB, our findings suggest that deep learning may serve as a complementary decision-support tool that enhances diagnostic confidence in the preoperative assessment of parotid gland tumors. Furthermore, the use of patient-level validation and histopathological confirmation for all cases strengthens the methodological rigor and clinical relevance of the present study.

The findings of the present study are consistent with the growing body of evidence supporting the application of deep learning in the diagnosis of parotid gland tumors. Recent MRI-based deep learning studies have demonstrated that convolutional neural network (CNN) models can achieve diagnostic performances comparable to, or under selected experimental conditions exceeding, those of conventional image interpretation for differentiating benign and malignant parotid lesions [9,10]. Likewise, multimodal deep learning approaches and radiomics-integrated models have further improved diagnostic classification by combining complementary imaging information with advanced feature extraction techniques [12,16,17].

In agreement with these previous studies, our results demonstrate that MRI-based deep learning can provide reliable diagnostic performance while maintaining a completely non-invasive workflow. Although the present study did not include a direct comparison with transformer-based architectures, internal benchmarking against the baseline ResNet50, DenseNet121, and EfficientNet-B0 models showed that the ResNet50+CBAM framework achieved the highest mean classification performance under the same experimental conditions. The component-level ablation comparison showed consistently higher mean accuracy, precision, recall, and F1-score after CBAM was added to the baseline ResNet50 under otherwise identical experimental conditions. These findings support the potential contribution of channel- and spatial-attention-based feature refinement, although the absence of formal statistical comparisons between architectures should be acknowledged. Nevertheless, direct comparisons with transformer-based and other contemporary architectures are required in future studies [18]. These findings suggest that careful image preprocessing, rigorous patient-level validation, and attention-guided feature extraction may contribute substantially to model performance, in addition to network architecture itself. Consistent with previous reports, the incorporation of the CBAM attention module likely enhanced the model’s ability to focus on diagnostically relevant image regions, thereby improving feature representation during classification [19]. Although the present model was developed using MRI images alone, future multimodal frameworks may provide a more comprehensive representation of parotid gland tumors. Recent studies combining clinical and imaging features also support the potential value of multimodal machine-learning approaches in salivary gland tumor classification [20]. Quantitative imaging parameters, particularly apparent diffusion coefficient values derived from diffusion-weighted imaging, may contribute information regarding tumor cellularity that is complementary to conventional morphological MRI features. The integration of ADC measurements with clinical characteristics, radiologic findings, and cytological information from FNAB may therefore improve diagnostic stratification, particularly in lesions with overlapping conventional imaging appearances. Future studies should evaluate whether such multimodal models provide additional patient-level diagnostic value beyond image-only deep learning.

By evaluating all three diagnostic approaches within the same clinical cohort, the present design provided a patient-level assessment of the potential complementary role of deep learning in the preoperative evaluation of parotid gland tumors.

Conventional MRI and FNAB remain indispensable components of the preoperative evaluation of parotid gland tumors. In the present study, the diagnostic performances of both modalities were consistent with those reported in previous investigations. MRI demonstrated high specificity and satisfactory sensitivity, confirming its established role in assessing tumor morphology, local extension, and the relationship of the lesion to adjacent anatomical structures [21,22]. Nevertheless, differentiating benign from malignant tumors solely on the basis of MRI findings remains challenging because of considerable overlap in imaging characteristics, particularly in lesions with atypical signal intensity or heterogeneous internal architecture [23].

Similarly, FNAB continues to be one of the most widely accepted diagnostic procedures owing to its high specificity, low cost, and minimally invasive nature [5,24]. However, its diagnostic accuracy may be influenced by sampling adequacy, tumor heterogeneity, and variability in cytopathological interpretation. In particular, false-negative findings in low-grade malignant tumors remain an important limitation that may affect preoperative diagnosis and subsequent surgical planning [25]. Taken together, these findings highlight that MRI and FNAB should be regarded as complementary rather than competing diagnostic modalities. Within this diagnostic framework, our results suggest that MRI-based deep learning may provide additional diagnostic support, particularly in clinically challenging cases where conventional imaging findings and cytological results are inconclusive, thereby improving diagnostic confidence without replacing established clinical practice.

Beyond its diagnostic performance, the proposed deep learning model has potential clinical implications for the preoperative evaluation of parotid gland tumors. Rather than functioning as a standalone diagnostic system, MRI-based artificial intelligence should be regarded as a decision-support tool that complements established diagnostic pathways. In routine clinical practice, such systems may assist radiologists and surgeons by providing standardized, reproducible, and observer-independent image interpretation, particularly in diagnostically challenging cases where MRI findings are equivocal or FNAB results are nondiagnostic or indeterminate. In this context, deep learning has the potential to improve diagnostic confidence, facilitate multidisciplinary decision-making, and enhance consistency in preoperative assessment without replacing clinical judgment [26,27,28].

Despite these promising findings, successful clinical implementation of artificial intelligence requires more than high diagnostic accuracy alone. Transparent model development, rigorous external validation, standardized reporting, and prospective clinical evaluation remain essential prerequisites before AI-assisted diagnostic systems can be incorporated into routine practice [26,27,28]. Therefore, artificial intelligence should currently be viewed as a complementary technology that supports, rather than replaces, experienced clinicians and established diagnostic methods. Within this framework, our findings provide further evidence that MRI-based deep learning can serve as an adjunctive tool for preoperative decision support in patients with parotid gland tumors, particularly when conventional imaging and cytological assessment do not provide a definitive diagnosis.

Several methodological strengths should be acknowledged when interpreting the findings of the present study. First, all diagnostic modalities were evaluated within the same patient cohort using histopathological diagnosis as the reference standard, enabling a direct and clinically meaningful comparison between MRI-based deep learning, conventional radiologic assessment, and FNAB. Second, model performance was assessed using strict patient-level cross-validation, thereby preventing data leakage between the training and validation datasets and providing a more realistic estimate of diagnostic performance. Third, all MRI examinations were interpreted by an experienced head and neck radiologist who was blinded to both cytological and histopathological findings, minimizing potential interpretation bias. Finally, Grad-CAM provided a qualitative visualization of the image regions contributing to model predictions. Although the activation maps showed partial correspondence with the tumor regions, they also included adjacent anatomical structures and should not be interpreted as precise lesion localization.

### Limitations

This study has several limitations. First, it was a retrospective single-center study, which may limit the generalizability of the proposed model to other institutions and patient populations. Second, the relatively small number of malignant tumors may have affected model performance for less common histopathological subtypes despite the use of subject-level cross-validation. The malignant class comprised only 29 independent patients, which represents a limited sample for training a high-capacity convolutional neural network. Although transfer learning, data augmentation, class weighting, early stopping, and strict subject-level five-fold cross-validation were used to reduce the risk of overfitting, these strategies cannot fully compensate for the limited number of independent malignant cases. Accordingly, the present findings should be interpreted as internally validated results that require confirmation in larger independent multicenter cohorts. In addition, because unequal numbers of images were selected from individual subjects to reduce image-level class imbalance, individual subjects contributed different numbers of images to model development. Although strict subject-level cross-validation prevented information leakage, this sampling strategy may have influenced the image-level performance estimates. Specifically, larger or more morphologically heterogeneous lesions may have contributed a greater number of informative images and therefore may have exerted greater influence during model training. In addition, image selection was performed manually by a single experienced radiologist using predefined anatomical and morphological criteria. No independent second-reader assessment or interobserver agreement analysis was performed. Therefore, the reproducibility of the image-selection process and the potential influence of reader-dependent selection bias could not be quantitatively assessed. Future studies should evaluate standardized multi-reader selection protocols or automated lesion segmentation and volume-based image sampling to improve reproducibility.

Third, the deep learning model was developed using MRI images alone; therefore, potentially relevant clinical and cytological variables were not incorporated into the prediction process. Although diffusion-weighted imaging was included in the imaging dataset, quantitative apparent diffusion coefficient measurements were not separately extracted or integrated into the model. Future studies should evaluate multimodal frameworks combining conventional MRI, quantitative diffusion parameters, clinical characteristics, radiologic findings, and FNAB results. The comparative benchmarking was limited to selected CNN architectures and did not include a transformer-based model. Therefore, the relative performance of the proposed framework against contemporary vision transformer architectures remains unknown.

Another important limitation is the lack of an independent external validation cohort. Although rigorous subject-level five-fold cross-validation was applied to minimize overfitting and prevent data leakage, the model was evaluated exclusively using internal cross-validation. As emphasized in recent methodological and reporting guidelines, independent multicenter validation is essential before artificial intelligence models can be considered for routine clinical implementation [27,28,29]. Federated learning may also represent a potential approach for multicenter model development in radiologic imaging [30].

Furthermore, Grad-CAM provided only qualitative visual explanations of the image regions contributing to model predictions. The activation maps were not quantitatively compared with radiologist-defined tumor boundaries and should not be interpreted as precise tumor localization or segmentation. Additional advances in explainable artificial intelligence and prospective clinical validation are required to improve clinician confidence and support the safe integration of AI-assisted diagnostic systems into clinical practice [31].

Finally, incomplete availability of certain baseline clinical variables, including tumor size, symptom duration, smoking history, and comorbidities, may have limited the assessment of potential confounding factors between benign and malignant tumors.

## 5. Conclusions

In this single-center cohort, the ResNet50+CBAM model demonstrated high patient-level diagnostic performance in differentiating benign and malignant parotid gland tumors. Its overall diagnostic performance was within the range observed for conventional MRI assessment and FNAB, supporting its potential role as a complementary decision-support tool rather than a replacement for established diagnostic methods. The direct comparison of deep learning, radiologic assessment, and FNAB within the same histopathologically confirmed patient cohort represents the principal clinical contribution of this study. However, the retrospective design, limited number of malignant cases, and absence of independent external validation restrict the generalizability of the findings. Prospective multicenter studies using external datasets are required before clinical implementation can be considered.

## Figures and Tables

**Table 1:** Demographic characteristics and dataset composition.

Variable	Value
**Tumor cohort demographics**	
Tumor cohort, total patients (*N*)	144
Age, mean ± SD (years)	44.9 ± 18.0
Age, median [IQR] (years)	48.0 [30.0–59.5]
Age range (years)	6–77
Sex: Male	80 (55.6%)
Sex: Female	64 (44.4%)
Side: Left	82 (56.9%)
Side: Right	62 (43.1%)
**Histopathological composition of the tumor cohort**	
Benign tumors	115 (79.9%)
Malignant tumors	29 (20.1%)
Pleomorphic adenoma	71 (49.3%)
Warthin tumor	44 (30.6%)
Mucoepidermoid carcinoma	23 (16.0%)
Adenoid cystic carcinoma	6 (4.2%)
**Deep learning dataset composition**	
Benign tumor subjects	115
Malignant tumor subjects	29
Normal control subjects	122
Total subjects included in the deep learning dataset	266
Original benign MRI images	780
Original malignant MRI images	528
Original control MRI images	734
Total original MRI images	2042

Note: Values are presented as *n* (%) unless otherwise specified. IQR, interquartile range; SD, standard deviation. MRI image counts represent original images obtained across multiple MRI sequences and anatomical levels and do not represent independent subjects or augmented samples. A greater number of original images was selected from malignant cases to reduce image-level class imbalance. All images belonging to the same individual were retained within a single cross-validation fold. Normal controls were included in the three-class deep learning analysis but were not included in the direct patient-level comparison of deep learning, MRI, and FNAB.

**Table 2:** Comparative and class-wise image-level performance of the evaluated CNN architectures.

A. Comparative Performance Across Five Validation Folds					B. Class-Wise Performance of ResNet50+CBAM				
Model	Accuracy, Mean ± SD	Precision, Mean ± SD	Recall, Mean ± SD	F1-Score, Mean ± SD	Class	Precision	Recall	F1-Score	ROC-AUC
ResNet50+CBAM	0.9716 ± 0.0127	0.9747 ± 0.0121	0.9723 ± 0.0153	0.9731 ± 0.0138	Benign	0.9617	0.9667	0.9642	0.99
ResNet50	0.86 ± 0.0207	0.74 ± 0.1081	0.80 ± 0.1086	0.76 ± 0.1021	Control	0.9649	0.9728	0.9688	1.00
DenseNet121	0.90 ± 0.0182	0.83 ± 0.1445	0.83 ± 0.1146	0.82 ± 0.1201	Malignant	0.9961	0.9773	0.9866	1.00
EfficientNet-B0	0.91 ± 0.0472	0.85 ± 0.1733	0.84 ± 0.1387	0.84 ± 0.1520					

Note: Panel A reports image-level macro-averaged performance as mean ± standard deviation across the five subject-level cross-validation folds. Panel B reports overall class-wise image-level metrics for the ResNet50+CBAM model. ROC-AUC values were calculated using a one-vs.-rest approach. The comparison between the baseline ResNet50 and ResNet50+CBAM represents a component-level ablation analysis evaluating the contribution of the CBAM. The ROC-AUC values in Panel B represent image-level one-vs.-rest performance and are distinct from the patient-level binary AUC values reported for the clinical comparison of MRI, FNAB, and deep learning.

**Table 3:** Diagnostic performance of MRI, FNAB, and the deep learning model compared with histopathology (patient-level analysis).

Parameter	MRI	FNAB	Deep Learning (ResNet50+CBAM)
True Positive (TP)	25	24	23
True Negative (TN)	107	111	108
False Positive (FP)	8	4	7
False Negative (FN)	4	5	6
Sensitivity	0.862	0.828	0.793 (95% CI: 0.616–0.902)
Specificity	0.930	0.965	0.939 (95% CI: 0.879–0.969)
Accuracy	0.917	0.938	0.910 (95% CI: 0.853–0.945)
Positive Predictive Value (PPV)	0.758	0.857	0.767 (95% CI: 0.588–0.882)
Negative Predictive Value (NPV)	0.964	0.957	0.947 (95% CI: 0.890–0.975)

Note: Deep learning metrics were calculated at the patient level using the 144 patients with histopathologically confirmed parotid tumors. A single patient-level prediction was obtained by majority voting across all selected MRI images belonging to each patient. Malignant tumors were considered the positive class. For the binary clinical analysis, benign and control predictions were grouped as non-malignant; therefore, benign tumors predicted as control were counted as non-malignant classifications. The separate cohort of 122 normal control subjects was excluded from the direct comparison with MRI and FNAB because these individuals were not members of the histopathologically confirmed tumor cohort. No patients had nondiagnostic or indeterminate FNAB results corresponding to Milan categories I, III, or IVb; therefore, all 144 tumor patients were included in the FNAB performance analysis. Confidence intervals were calculated using the Wilson score method.

## Data Availability

The data presented in this study are available on request from the corresponding author due to patient privacy and ethical restrictions.

## Supplementary Materials

The following supporting information can be downloaded at https://www.mdpi.com/article/10.3390/diagnostics16162577/s1, Table S1. Diagnostic performance of MRI, FNAB, and deep learning model compared with histopathology.

## References

[1] Speight P.M., Barrett A.W. (2002). Salivary gland tumours. Oral Dis..

[2] Guzzo M., Locati L.D., Prott F.J., Gatta G., McGurk M., Licitra L. (2010). Major and minor salivary gland tumors. Crit. Rev. Oncol. Hematol..

[3] Christe A., Waldherr C., Hallett R., Zbaeren P., Thoeny H. (2011). MR imaging of parotid tumors: Typical lesion characteristics in MR imaging improve discrimination between benign and malignant disease. AJNR Am. J. Neuroradiol..

[4] Liu C.C., Jethwa A.R., Khariwala S.S., Johnson J., Shin J.J. (2016). Sensitivity, specificity, and posttest probability of parotid fine-needle aspiration: A systematic review and meta-analysis. Otolaryngol. Head Neck Surg..

[5] Colella G., Cannavale R., Flamminio F., Foschini M.P. (2010). Fine-needle aspiration cytology of salivary gland lesions: A systematic review. J. Oral Maxillofac. Surg..

[6] Eida S., Sumi M., Sakihama N., Takahashi H., Nakamura T. (2007). Apparent diffusion coefficient mapping of salivary gland tumors: Prediction of the benignancy and malignancy. Am. J. Neuroradiol..

[7] Coudert H., Mirafzal S., Dissard A., Boyer L., Montoriol P.F. (2021). Multiparametric magnetic resonance imaging of parotid tumors: A systematic review. Diagn. Interv. Imaging.

[8] Litjens G., Kooi T., Bejnordi B.E., Setio A.A.A., Ciompi F., Ghafoorian M., van der Laak J.A.W.M., van Ginneken B., Sánchez C.I. (2017). A survey on deep learning in medical image analysis. Med. Image Anal..

[9] Xia X., Feng B., Wang J., Hua Q., Yang Y., Sheng L., Mou Y., Hu W. (2021). Deep learning for differentiating benign from malignant parotid lesions on MR images. Front. Oncol..

[10] Liu X., Pan Y., Zhang X., Sha Y., Wang S., Li H., Liu J. (2023). A deep learning model for classification of parotid neoplasms based on multimodal magnetic resonance image sequences. Laryngoscope.

[11] Mai W., Fan X., Zhang L., Li J., Chen L., Hua X., Zhang D., Li H., Cai M., Shi C. (2025). Deep learning for differential diagnosis of parotid tumors based on 2.5D magnetic resonance imaging. Ann. Med..

[12] Wang Y., Gao J., Yin Z., Wen Y., Sun M., Han R. (2024). Differentiation of benign and malignant parotid gland tumors based on the fusion of radiomics and deep learning features on ultrasound images. Front. Oncol..

[13] Rossi E.D., Faquin W.C., Baloch Z., Barkan G.A., Foschini M.P., Pusztaszeri M., Vielh P., Kurtycz D.F.I. (2017). The Milan System for Reporting Salivary Gland Cytopathology: Analysis and suggestions of initial survey. Cancer Cytopathol..

[14] Woo S., Park J., Lee J.Y., Kweon I.S. (2018). Cbam: Convolutional block attention module. Proceedings of the European Conference on Computer Vision (ECCV).

[15] Theckedath D., Sedamkar R.R. (2020). Detecting affect states using VGG16, ResNet50 and SE-ResNet50 networks. SN Comput. Sci..

[16] Xia Z., Huang X.C., Xu X.Y., Miao Q., Wang M., Wu M.J., Zhang H., Jiang Q., Zhuang J., Wei Q. (2025). Ultrasound-Based Deep Learning Radiomics Models for Predicting Primary and Secondary Salivary Gland Malignancies: A Multicenter Retrospective Study. Bioengineering.

[17] Khodabakhshi Z., Motisi L., Bink A., Broglie M.A., Rupp N.J., Fleischmann M., von der Grün J., Guckenberger M., Tanadini-Lang S., Balermpas P. (2024). MRI-based radiomics for predicting histology in malignant salivary gland tumors: Methodology and “proof of principle”. Sci. Rep..

[18] Zhang G., Zhu L., Huang R., Xu Y., Lu X., Chen Y., Li C., Lei Y., Luo X., Li Z. (2023). A deep learning model for the differential diagnosis of benign and malignant salivary gland tumors based on ultrasound imaging and clinical data. Quant. Imaging Med. Surg..

[19] Sinci K.A., Koska I.O., Cetinoglu Y.K., Erdogan N., Koc A.M., Eliyatkin N.O., Koska C., Candan B. (2025). Deep learning-based classification of parotid gland tumors: Integrating dynamic contrast-enhanced MRI for enhanced diagnostic accuracy. BMC Med. Imaging.

[20] Su H.Z., Li Z.Y., Hong L.C., Wu Y.H., Zhang F., Zhang Z.B., Zhang X.D. (2025). Machine learning model for diagnosing salivary gland adenoid cystic carcinoma based on clinical and ultrasound features. Insights Imaging.

[21] Zheng Y.M., Li J., Liu S., Cui J.F., Zhan J.F., Pang J., Zhou R.Z., Li X.L., Dong C. (2021). MRI-Based radiomics nomogram for differentiation of benign and malignant lesions of the parotid gland. Eur. Radiol..

[22] Assili S., Fathi Kazerooni A., Aghaghazvini L., Saligheh Rad H.R., Pirayesh Islamian J. (2015). Dynamic Contrast Magnetic Resonance Imaging (DCE-MRI) and Diffusion Weighted MR Imaging (DWI) for Differentiation between Benign and Malignant Salivary Gland Tumors. J. BioMed. Phys. Eng..

[23] Kim S.Y., Borner U., Lee J.H., Wagner F., Tshering Vogel D.W. (2022). Magnetic resonance imaging of parotid gland tumors: A pictorial essay. BMC Med. Imaging.

[24] Schmidt R.L., Hall B.J., Wilson A.R., Layfield L.J. (2011). A systematic review and meta-analysis of the diagnostic accuracy of fine-needle aspiration cytology for parotid gland lesions. Am. J. Clin. Pathol..

[25] De Ru J.A., van Benthem P.P. (2023). Diagnostic accuracy of fine-needle aspiration cytology in parotid gland lesions: A review. Acta Otolaryngol..

[26] Zhang R., Wong L.M., So T.Y., Cai Z., Deng Q., Tsang Y.M., Ai Q.Y.H., King A.D. (2024). Deep learning for the automatic detection and segmentation of parotid gland tumors on MRI. Oral Oncol..

[27] Collins G.S., Moons K.G.M., Dhiman P., Riley R.D., Beam A.L., Van Calster B., Ghassemi M., Liu X., Reitsma J.B., van Smeden M. (2024). TRIPOD+AI statement: Updated guidance for reporting clinical prediction models that use regression or machine learning methods. BMJ.

[28] Park S.H., Suh C.H. (2024). Reporting Guidelines for Artificial Intelligence Studies in Healthcare (for Both Conventional and Large Language Models): What’s New in 2024. Korean J. Radiol..

[29] Yu A.C., Mohajer B., Eng J. (2022). External Validation of Deep Learning Algorithms for Radiologic Diagnosis: A Systematic Review. Radiol. Artif. Intell..

[30] Rehman M.H.U., Hugo Lopez Pinaya W., Nachev P., Teo J.T., Ourselin S., Cardoso M.J. (2023). Federated learning for medical imaging radiology. Br. J. Radiol..

[31] Houssein E.H., Gamal A.M., Younis E.M.G., Mohamed E. (2025). Explainable artificial intelligence for medical imaging systems using deep learning: A comprehensive review. Clust. Comput..

