# Supplementary material for: Deep Learning-Assisted MRI for Differentiating Parotid Gland Tumors: Comparison with FNAB and Radiologic Assessment
Source: Diagnostics (Basel). 2026 Aug 15;16(16):2577. doi: 10.3390/diagnostics16162577 (PMC13512347; doi:10.3390/diagnostics16162577)
Supplement: Supplementary file 1 [file diagnostics-16-02577-s001.zip › diagnostics-4511290-supplementary.pdf]

Table S1. Diagnostic performance of MRI, FNAB, and deep learning model compared with histopathology

Parameter	MRI	FNAB	Deep Learning (ResNet50 + CBAM)
True Positive (TP)	24	24	258
True Negative (TN)	108	110	377
False Positive (FP)	8	4	1
False Negative (FN)	4	6	5
Sensitivity	0.862	0.828	0.981
Specificity	0.930	0.965	0.997
Accuracy	0.917	0.938	0.991
Positive Predictive Value (PPV)	0.758	0.857	0.996
Negative Predictive Value (NPV)	0.964	0.957	0.987

FNAB shows high specificity (96.5%) but relatively moderate sensitivity (82.8%), consistent with classical cytological performance in parotid tumors. MRI achieves slightly better sensitivity (86.2%) but lower PPV, suggesting more false positives than FNAB. The deep learning model markedly outperforms both, with accuracy above 99%, sensitivity 98%, and specificity 99.7%, indicating strong potential as a noninvasive diagnostic adjunct.(table S1)
